# Proteinase 3; a potential target in chronic obstructive pulmonary disease and other chronic inflammatory diseases

**DOI:** 10.1186/s12931-018-0883-z

**Published:** 2018-09-20

**Authors:** Helena Crisford, Elizabeth Sapey, Robert A. Stockley

**Affiliations:** 1 Institute of Inflammation and Ageing, University of Birmingham, Edgbaston, Birmingham, B15 2GW UK; 2 University Hospital Birmingham NHS Foundation Trust, Edgbaston, Birmingham, B15 2GW UK; 3 Institute of Inflammation and Ageing, College of Medical and Dental Sciences, Centre for Translational Inflammation Research, University of Birmingham Research Laboratories, Queen Elizabeth Hospital Birmingham, Mindelsohn Way, Birmingham, B15 2WB UK

**Keywords:** Proteinase 3/myeloblastin, Serine proteinases, Chronic obstructive pulmonary disease, Lungs, Inflammation

## Abstract

Chronic Obstructive Pulmonary Disease (COPD) is a common, multifactorial lung disease which results in significant impairment of patients’ health and a large impact on society and health care burden. It is believed to be the result of prolonged, destructive neutrophilic inflammation which results in progressive damage to lung structures. During this process, large quantities of neutrophil serine proteinases (NSPs) are released which initiate the damage and contribute towards driving a persistent inflammatory state.

Neutrophil elastase has long been considered the key NSP involved in the pathophysiology of COPD. However, in recent years, a significant role for Proteinase 3 (PR3) in disease development has emerged, both in COPD and other chronic inflammatory conditions. Therefore, there is a need to investigate the importance of PR3 in disease development and hence its potential as a therapeutic target. Research into PR3 has largely been confined to its role as an autoantigen, but PR3 is involved in triggering inflammatory pathways, disrupting cellular signalling, degrading key structural proteins, and pathogen response.

This review summarises what is presently known about PR3, explores its involvement particularly in the development of COPD, and indicates areas requiring further investigation.

## Background

The serine proteinase Proteinase 3 (PR3) is an enzyme released during neutrophilic inflammation and is capable of cleaving many targets including key structural proteins of the lung. Chronic Obstructive Pulmonary Disease (COPD) is an inflammatory condition associated with neutrophilic inflammation. For this reason neutrophil elastase (NE) has long been considered to be a central, proteinase in the pathophysiology as it can replicate many of the structural changes of the disease and hence a potential target for therapeutic manipulation, PR3,another key neutrophil serine proteinase has largely been ignored, even though it may have an important additional role in the lung as well as other human diseases [1]. This review summarises the current literature to provide an update on the potential role of PR3 in health and disease, with a primary focus on COPD.

## Proteinase 3

PR3, alternatively referred to as myeloblastin, azurophil granule protein-7 or p29b, is a highly abundant neutrophil protein which is genetically transcribed in primitive myeloid and monocytic progenitor cells, and expressed in cells of granulocyte and monocyte linage, especially neutrophils but including mast cells and basophils [2–5] and in the neutrophil, it is mainly located within the primary azurophil granules of the mature cell but is also present in specific granules, secretory vesicles, and on the cell surface [6, 7]. It is expressed constitutively on the membrane by naïve neutrophils in peripheral blood of healthy individuals (known as “constitutive” PR3) and is secreted into extracellular medium by activated neutrophils following granule translocation to the cell membrane (known as “induced” PR3) [8–11].

It is encoded by the gene *PRTN3* which is located at human chromosome 19p13.3 and spans 6.57 kb pairs including 5 exons and 4 introns. The gene consists of 222 amino acids that fold to form the 29 kDa glycoprotein PR3 [4].

**Fig. 1:**
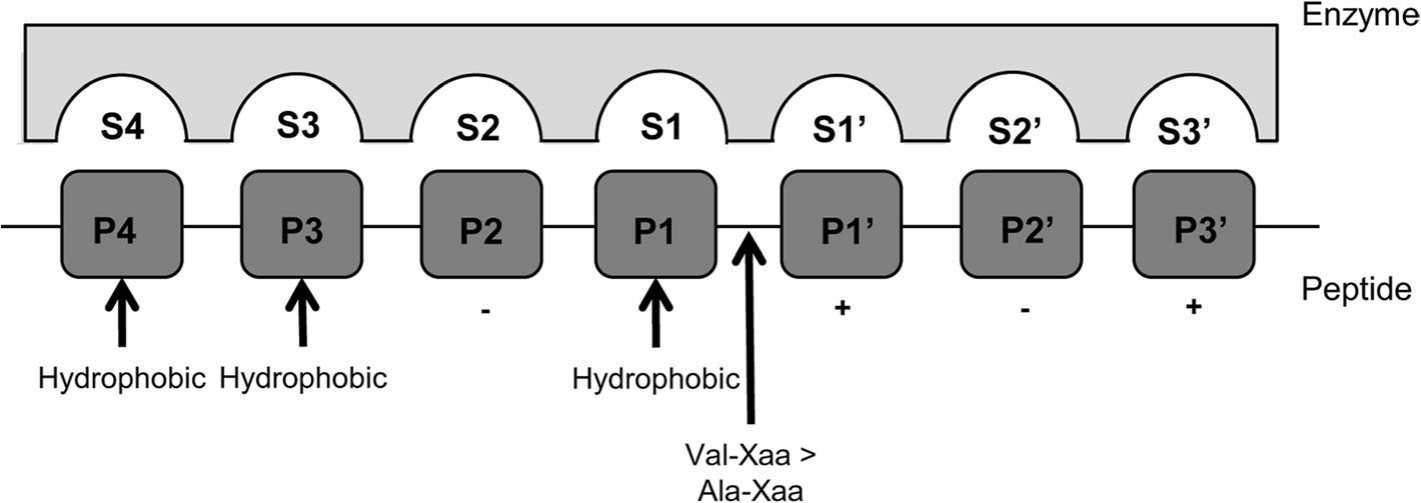
Diagrammatically demonstrates the substrate binding pockets S4-S3’ of PR3 with substrate cleavage positions P4-P3’, according to the Schechter and Berger enzyme-ligand binding site numbering convention [19]. The arrows indicate the sites for Val/Ala-containing peptide cleavage and hydrophobic residue binding sites, whilst + indicates positive and – indicates negative residue binding site. Adapted from [13]

PR3 is classified within the family of “chymotrypsin”-like neutrophil serine proteinase (NSP) which are identified by their highly conserved catalytic triads (His57, Asp102 and Ser195; using chymotrypsinogen numbering) for proteolytic activity and defined by their active site serine residue [4, 12]. PR3 possesses an enlarged binding site with high specificity and differs from NE by 4 main subsites, S2, S1’, S2’ and S3’ (Fig. 1). which is common to other NSPs, including NE [12]. However, PR3, specificity is further defined by difference in residues which alter subsite specificities (subsites shown in Fig. 1).

These specificities are determined by:

- Amide hydrogens on Gly193 and Ser195 which stabilise charge during catalysis [14].
- 3 charged residues: Lys99, Asp61, Arg143 within the active site region.
- Positioning of the solvent accessible Lys99 (compared to Leu99 in NE), which borders the S2 and S4 sites and makes the S2 subsite deeper and more polar, in addition to reducing its hydrophobicity, which determines preferential binding of negative and polar residues, such as Asp [12, 16, 17].
- Asp61 brings the proteins negatively charged side chain closer to the S1’ and S’3 subsites, making the subsites smaller and more polar, which encourages binding of basic residues at P1’ and P’3 [12, 16].
- Arg143 (and Pro151) increase the polarity of the S2’ subsite which creates a basic S2’ subsite that binds acidic residues [12, 16].
- Asp213 (compared to Ala213 in NE) restricts the S1 binding site causing it to preferably bind small hydrophobic residues at P1, which includes alanine, serine, valine, norvaline, and methionine [12, 14, 16–18].
- Ile217 allows small hydrophobic residues at P4 to bind whilst with Trp218 creating a more hydrophobic S5 subsite [12, 14, 16].

**Fig. 2:**
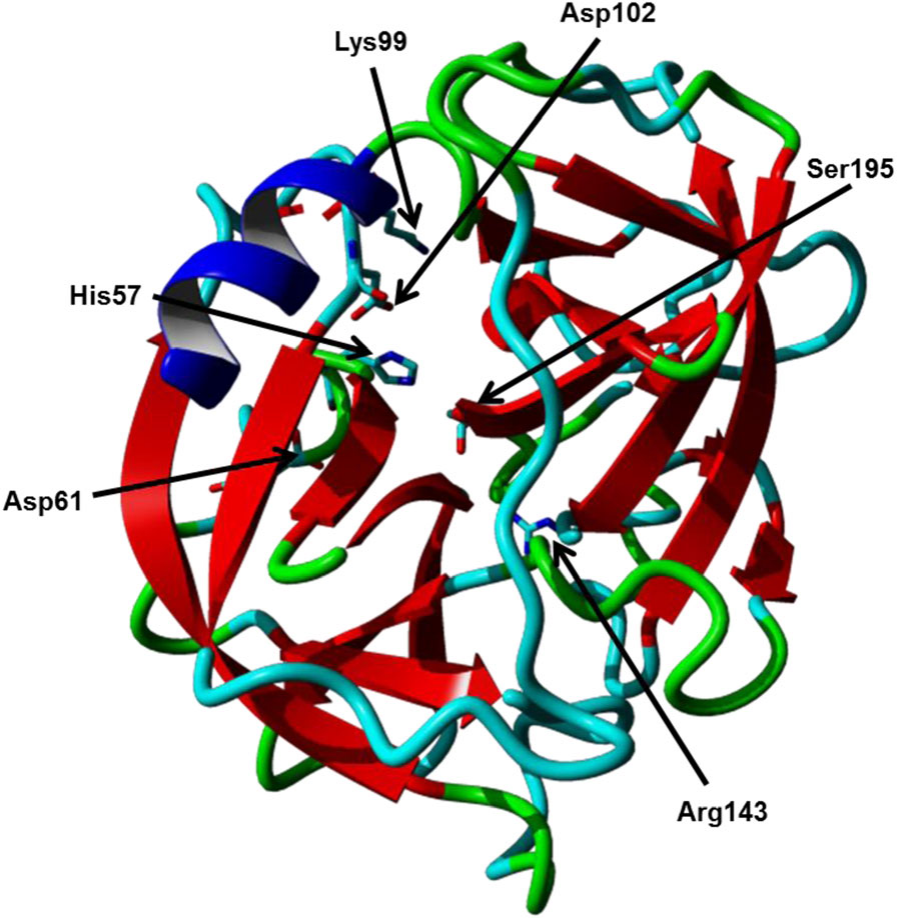
Three-dimensional visualisation of Proteinase 3 by ribbon plot, with the catalytic triad and PR3-specific residues stylised in a stick representation and annotated. Image developed from the Proteinase 3 Protein Data Bank entry (PDB ID: 1FUJ) [15, 21] using YASARA [22]

PR3 is initially transcribed as an inactive precursor referred to as a zymogen and then undergoes a two-stage posttranslational modification to become active. Firstly (via signal peptidase), there is N-terminal signal peptide cleavage, followed by cleavage of the N-terminal pro-di-peptide by the cysteine proteinase, cathepsin C which is essential for enzymatic activity. Secondly it undergoes pro-peptide cleavage at the C terminal, which is crucial for granule packaging. This forms the catalytic triad of residues and the final conformation of mature PR3, as shown in Fig. 2, which is stabilised by disulphide bonds and appropriate asparagine-linked glycosylation [4, 12, 20]. PR3 then remains stored within the neutrophil azurophil granules until release.

There is estimated to be 3 pg of PR3 stored in each neutrophil, alongside other key serine proteinases, with a mean PR3 concentration of 13.4 mM in each granule, which is 3–4 fold higher than NE [23]. Once released, either constitutively or via granule translocation, PR3 can act enzymatically in both an intracellular and extracellular manner. Its activity is then controlled through inactivation by inhibitors (both reversibly and irreversibly), including serine proteinase inhibitors (Serpins), chelonianin inhibitors and also alpha-2-macroglobulin [24–26].

PR3 has many functions. Animal transgenic and knockout models have demonstrated that it is able to cleave structural proteins leading to tissue remodelling (as discussed within ‘Pathophysiological functions of PR3 in COPD’), through diffusing deeper into tissues than the other NSPs [27–30]. Other functions assist in the defensive immune role of the neutrophil including regulating a variety of cellular processes, cleaving host protein into antibacterial peptides and activating pro-inflammatory cytokines [31, 32]. Dysfunction of these systems has long been associated with the development or progression of a number of chronic inflammatory diseases including COPD, but often without reference to the potential role of PR3.

## Pathophysiological functions of proteinase 3 in COPD

PR3 is likely to have more involvement in the pathophysiology of COPD than previously thought, supported by evidence of increased PR3 activity described in these patients [33–35]. COPD is a progressive, destructive lung disease associated with chronic neutrophilic inflammation and marked by obstruction of airflow, reduced physical activity and breathlessness [36].

The pathophysiology of COPD is considered to reflect an imbalance between proteinases and anti-proteinases in the lung, and Sinden et al. produced the first substantive evidence to support the role of PR3 in a three-dimensional reaction diffusion lung interstitium model [27]. The authors demonstrated that active proteinase diffusion distance following release from a neutrophil varies predominantly depending on concentrations of local physiological inhibitors and that this was greater for PR3 than NE [27]. Generally, activated proteinases have the potential to cause direct lung damage, whereas anti-proteinases provide protection to limit this process. In the lungs, a homeostatic balance is largely maintained, with the exception of a region of quantum proteolysis surrounding migrating and degranulating neutrophils which is larger in patients with α-1 anti-trypsin deficiency (AATD) explaining the increased susceptibility of these subjects to COPD [37]. This reflects the high concentrations of NSPs released from the granules compared to the immediate concentration of the physiological inhibitors. As the NSPs diffuse away from the neutrophil, the concentration falls exponentially until it equals that of the surrounding inhibitors when activity ceases [37]. It is believed that when levels of NSPs, including PR3, exceed the amount of protective anti-proteinases, such as α-1 anti-trypsin (AAT), excessive damage to lung tissue and other proteinase effects are facilitated [38].

It was previously believed that NE played the key neutrophilic role in tissue damage leading to emphysema, especially in subjects with genetic deficiency of AAT. However, recent data has challenged this concept and supports a potential role for other NSPs including PR3 [33]. Firstly, when a migrating neutrophil degranulates in vitro, it is expected to release more PR3 than NE from the azurophil granules. In vitro *s*ome of this becomes membrane bound and more resistant to inhibition, also the free enzyme still has a far greater radius of activity than NE [10, 39, 40]. In addition, although local lung-derived inhibitors are able to inhibit NE the same is not true for PR3, and persistent activity is detectable in lung secretions when NE activity is not [41, 42]. This is important as it implies all the pathological changes in the lung attributed to NE may also be produced by PR3 and potentially to a greater extent.

This theory is supported in vivo by the development of emphysema in hamsters receiving local administration of PR3 and further by recent evidence that SerpinA1-deficient murine models develop spontaneous emphysema [43, 44]. In addition, NSP-knockout murine models are protected against developing emphysema induced by cigarette smoke, whereas mice only deficient in NE are less susceptible, implying that either cathepsin G or PR3 played an important role [30]. Collectively these models suggest that, as well as NE, PR3 is potentially able to contribute to the development of emphysema in humans.

Biochemical studies have shown that PR3 cleaves extracellular matrix (ECM) proteins, including elastin, fibronectin, vitronectin, laminin and collagen, at a GXXPG site within a β-fold conformation resulting in protein degradation [17, 45, 46]. These proteins are important components of tissue structures and, it is the degradation of the extracellular matrix which results in the connective tissue injury in the lung interstitium leading to emphysema, as observed using biomarkers in human COPD and as induced in several animal models [43, 46–48]. Indeed, recent evidence suggests PR3 specific cleavage of elastin is elevated in COPD providing more direct evidence of its role [33].Like other NSPs, PR3 can also affect mucus clearance by damaging bronchial epithelium and cilia [16]. In addition, PR3 is able to induce mucus production from submucosal gland serous cells and PR3 activity has been implicated in this role in cystic fibrosis (CF) [49]. The net result is excess mucus production in the airways and impaired mucus clearance, which is also a feature of chronic bronchitis, and therefore PR3 is likely to have a similar role in COPD. AATD is a genetic cause of emphysema and chronic bronchitis (in about 30% of patients) and is the result of mutations resulting in little/no production of functional AAT protein. PR3 has a lower association rate with AAT than NE, which means that, in patients with AATD, PR3 is even more poorly regulated, causing a greater proteinase/anti-proteinase imbalance than with NE, and hence potentially mediates more damage to the lungs [4, 27, 50].

As well as causing direct tissue damage, PR3 is also potentially involved in amplifying the inflammation associated with COPD as with other chronic inflammatory diseases.

PR3 is known to modulate a variety of cytokine functions, which impact processes such as metabolism and inflammasome generation [51–53]. The enzyme facilitates an increased production and/or modulation of proinflammatory cytokines and the reduction of anti-inflammatory cytokine production as summarised in Table 1. Many of these cytokines have been implicated in a number of inflammatory diseases, which supports a putative role of PR3 in chronic inflammatory conditions in general as well as COPD with and without AATD.

**Table 1:** Summary of the cytokines affected by PR3, with the PR3 action on cytokines and the resulting response. The processes relevant to the pathophysiology of COPD are highlighted in bold

Cytokine	Role of PR3	Action of cytokine	References
Interleukin (IL)-1β	Proteolytically activates extracellular pro-forms to be cleaved into active counterparts by Caspase 1 in inflammasomes	• **↑ neutrophil activation and recruitment** • **canonical NFκB signalling** • ↑ cyclooxygenases [44] and prostaglandin E production • pushes towards T helper cell (Th)17 differentiation	[31, 54–56]
IL-18		• Induces interferon (IFN)-γ and Fas ligand, ↑ differentiation to Th1, Th2 or Th17 responses (dependant on accompanying signals)	[55, 57]
Tumour necrosis factor (TNF)-α	Cleaves precursor to bioactive form (via two hypothesised cleavage sites at Ala15-Leu16 or Val77-Arg78)	• **Activates the caspase and MEK cascades, and PI-3-kinase and canonical NFκB pathway** • **Activates Etk = ↑cellular adhesion, migration and propagation** • **↑ neutrophil chemotaxis** • **Upregulation of pro-inflammatory genes e.g. IL-8, CCL2, CXCL10, COX-2, and pro-coagulants** • **Recruits apoptosis-inhibiting molecules** • ↓ signalling by cIAP-mediated ubiquitination	[54, 58]
IL-6	Functionally inactivates and degrades the soluble IL-6 receptor (sIL-6R) – exact mechanisms unknown	• Disrupts trans-signalling activity • **Prevents apoptosis** • **↑ neutrophil recruitment and infiltration**	[59, 60]
IL-8 (CXCL8)	Truncates stored IL-8 (77) into the 10-fold more potent chemo-attractant IL-8 (70) through cleavage of an Ala-Lys bond	• **↑ respiratory burst** • **Potentiates inflammatory disease cycle** • **Drives neutrophil chemotaxis**	[61]
IL-17 (CTLA8)	Stimulation increases cytokine production	• Directs towards a dominant Th17 environment • T cell hypo-responsiveness	[62]
IL-32	Processes activating cytokines IL-1β, TNF-α and IFN-γ directly or indirectly; cleaves IL-32 at IL-32α to a more bioactive form	• **Activates canonical NFκB and MAPK cascades** • **↑ production of cytokines incl. TNF-α, IL-8 and CXCL2 production**	[63, 64]

All these cytokines can act through autocrine, paracrine and endocrine pathways to activate pro-inflammatory cascade responses and upregulate pro-inflammatory genes and transcription factors leading to an inflammatory state [65]. The products of these key inflammatory pathways can further induce feedback loops to enhance chronic inflammation [66–68]. Therefore (similarly to NE) PR3 can potentially play multiple roles in the initiation and amplification as well as the resolution of inflammation, at least as demonstrated in vitro.

More recently, PR3 has been also found to degrade the anti-inflammatory mediator progranulin (PGRN), resulting in generation of granulin (GRN) peptides in vitro [32, 68–70]. PGRN degradation causes increased neutrophil infiltration, activation of reactive oxidative species, pro-inflammatory cytokine production and anti-inflammatory pathway inhibition, sustaining an inflammatory state in other inflammatory disease [71]. GRN molecules are also known to accumulate and release the chemoattractant interleukin (IL)-8 amplifying neutrophil recruitment [70, 72]. In clinically-stable COPD, the concentration of PR3 in airway secretions is a stronger predictor of PGRN levels than NE, because of its greater neutrophil concentration and hence greater secretion activity [69].

PR3 is also able to act in a pro-inflammatory manner by interacting with the complement pathway. It is able to fragment the neutrophil surface complement component 5a (C5a) receptor, resulting in the loss of the N-terminus and an inability to bind C5a [73]. In CF, the lack of C5aR signalling contributes towards inefficient clearance of microbial infections in vitro and also inactivates signalling and stimulates neutrophils to degranulate [73]. This results in a cycle of dysfunctional neutrophils thereby perpetuating the bacterial-stimulated inflammatory signals and further neutrophil recruitment. Although there is no direct evidence, it is likely that C5aR inhibition by PR3 also has a role in COPD with elevated levels of C5a in the sputum of patients and correlations with circulating C5a, physiological gas transfer and the degree of emphysema [74]. Further research is clearly indicated to determine the relevance of this mechanism in COPD.

Despite the potential to impede bacterial clearance, it has also been reported that PR3 itself possesses bactericidal properties through cleavage of the pro-microbicidal protein hCAP-18 (human cathelicidin) into the antibacterial peptide, mucus inducer and neutrophil chemo-attractant LL-37 [51, 75–78]. Furthermore, levels of LL-37 in sputum are related to disease severity in patients with COPD suggesting an indirect role for PR3 which is worthy of further investigation [79].

In addition, PR3 can adhere to neutrophil extracellular traps (NETs) contributing towards the destruction of bacterial virulence factors [80–82]. However, many respiratory-relevant bacteria, such as *Streptococcus pneumoniae* and *Haemophilus influenzae,* have evolved NET evasion mechanisms which may overcome this potential clearance mechanism [83, 84]. It has also been noted that patients with *Pseudomonas aeruginosa* infection are more susceptible to poor outcome when lacking sufficient PR3 inhibition and patients with AATD are at particularly high risk of respiratory infection and lung damage as other natural proteinase inhibitors are unable to compensate for low AAT levels [27, 85]. This is again amplified by the greater neutrophil PR3 content and the fact that the other major lung inhibitor of serine proteinases, secretory leukocyte proteinase inhibitor (SLPI), does not inhibit PR3 [86].

However, PR3 is also able to inactivate SLPI, by cleaving at the Ala-16 site within the N-terminal and preventing SLPI/enzymes complex formation which would indirectly amplify the local activity of other serine proteinases such as NE [86].

Analysis of biopsied lung tissue, from patients with severe emphysema, has shown that cytosolic PR3 interrupts the initiation of anti-inflammatory mechanisms and promotes an apoptotic environment, inducing death of lung epithelial cells which has been implicated in the pathophysiology of emphysema by a further indirect route [87].

An additional mechanism implicated in the pathophysiology of COPD involves the receptor for advanced glycation end-products (RAGE) and soluble RAGE. In prostate cancer cell lines, PR3 has been shown to bind to RAGE both promoting cell activation and preventing its cleavage which escalates inflammation [88, 89]. Furthermore, decreased levels of sRAGE have been implicated in emphysema development [89, 90]. Clearly the relevance of this alternative function also needs to be explored in relation to COPD.

## Pathophysiological functions of proteinase 3 in other diseases

**Fig. 3:**
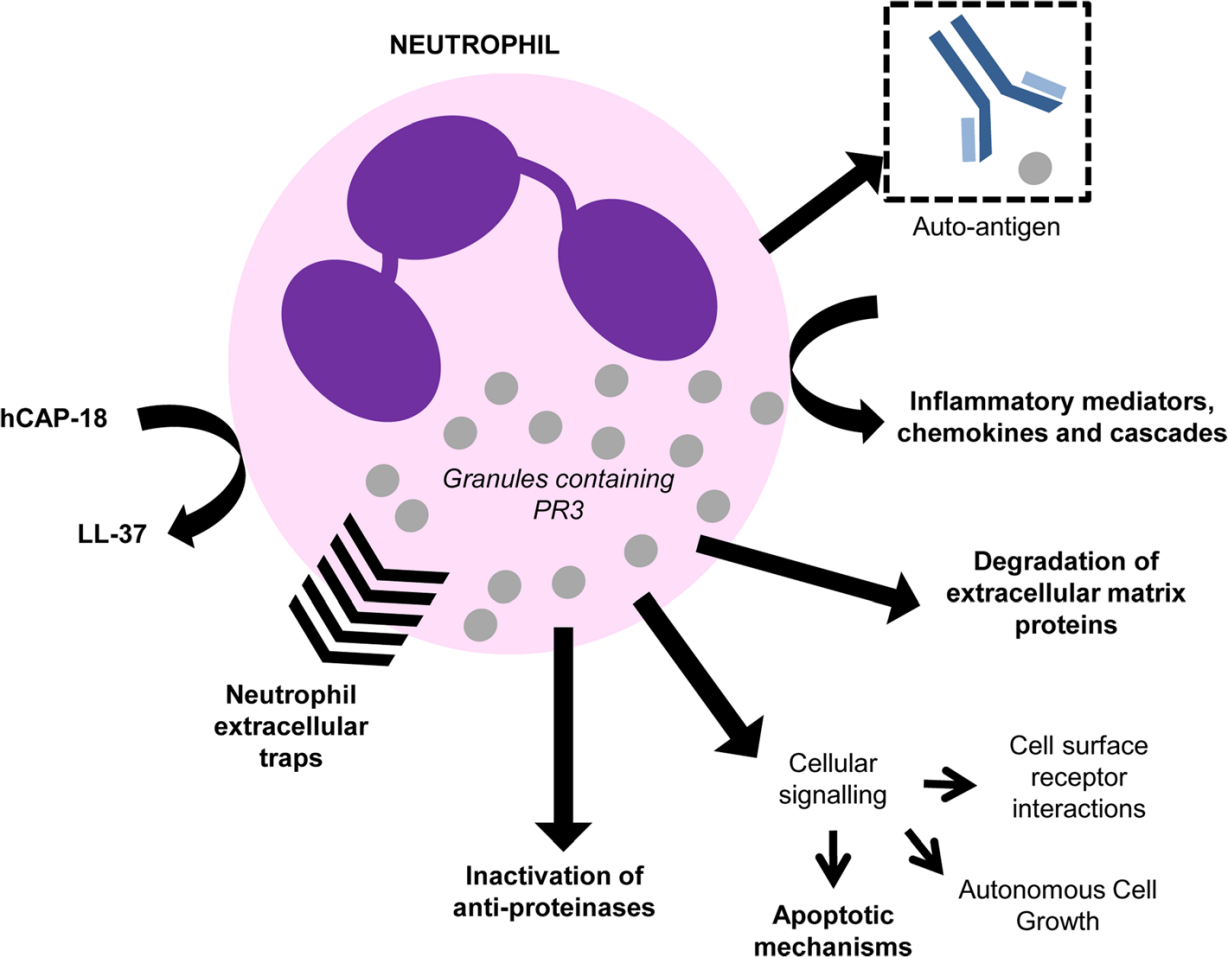
Summary of the actions of Proteinase 3 (PR3), as outlined in this review, which likely impact on COPD and other systemic diseases. The processes with a putative central role in the pathophysiology of emphysema are highlighted in bold

The actions discussed above are not just relevant to COPD but are relevant to the pathophysiology of many other diseases. PR3 also has many additional roles which can lead to, or amplify other disease states (see Fig. 3).

As noted in Table 1, PR3 has both a direct and indirect effect on many cytokines and hence can have further downstream influences on diseases beyond or associated with COPD, as outlined in Table 2.

**Table 2:** Cytokines influenced by PR3 (as shown in Table 1) and implicated in disease states other than COPD

Cytokine	Diseases Implicated	References
Interleukin (IL)-1β	• Rheumatoid arthritis • Asthma	[91, 92]
IL-18	• Non-alcoholic fatty liver disease • Type 2 diabetes • Asthma • Rheumatoid arthritis	[92–94]
Tumour necrosis factor (TNF)-α	• Rheumatoid arthritis • Interstitial Lung Disease • Asthma	[95, 96]
IL-6	• Cystic fibrosis	[97]
IL-17 (CTLA8)	• Granulomatosis with polyangiitis	[62]
IL-32	• Psoriasis • Rheumatoid arthritis • Crohn’s disease	[98]

However, although the effects of dysregulation of these cytokines are also implicated in other diseases, PR3 has not been directly studied in relation to their pathophysiology.

In addition, the interaction between PR3 and PGRN also likely has wider impact than in COPD, through a further role in inflammatory conditions involving PGRN, including lipopolysaccharide-induced acute lung injury, dermatitis and inflammatory arthritis (in murine models), as well as a reported genetic link between loss-of-function mutations in PGRN and the development of neurodegenerative disease [99–104].

It is also suspected that PR3, alongside other NSPs, could have a role in ECM breakdown affecting the pathophysiology of diseases in other organs, such as aneurysms due to vascular remodelling as shown in porcine vasculature; however the relevance has not yet been investigated in detail in humans [105].

PR3 has a role in the efficacy of neutrophil transmigration through interaction with the cell surface receptor NB1 (CD177) which acts with PECAM-1 (CD31) during trans-endothelial migration of neutrophils [106, 107]. In CF, this is supported by a positive relationship between PR3 activity and neutrophil migration effectiveness [20]. The interaction of PR3 with NB1 and PECAM-1 is confirmed in vitro in endothelial cells, where it inhibits activation and upregulation of these adhesion molecules [108].

There is also evidence that PR3 is associated with distortion of cellular signalling pathways and the development of autonomous cell growth. In leukaemia, early expression of PR3 during haematopoiesis is able to induce factor-independent growth and overexpression of PR3 in myeloid leukaemia cells prevents their differentiation into monocytoid cells supporting this mechanism [109–111].

Alternatively to its pro-apoptotic role in COPD, PR3 may paradoxically prevent apoptosis in granulomatosis with polyangiitis (GPA) by associating with calreticulin, through co-externalisation with phosphatidylserine by phospholipid flip-flop via phospholipid scramblase 1 (PLSCR1), to override the ‘eat me’ signalling [112].

Finally, PR3 also has a role as an autoantigen in many diseases, including GPA and idiopathic interstitial pneumonias, and is the target of cytoplasmic (c)-anti-neutrophil cytoplasmic antibodies, also referred to as PR3-ANCA in vasculitis [113–115]. Development of disease is dependent on ability of PR3 to associate with the cell membrane [112]. The binding of PR3-ANCA with cell associated PR3 initiates a cascade which amplifies inflammation and results in local cellular and tissue damage [9, 114, 116–118].

It was suspected that PR3-ANCA formation may have a role in COPD development, as more patients with COPD were found to be antinuclear antibody positive than healthy controls [119]. However, despite a reported association with emphysema-dominant disease and lower body mass index, no clear pathophysiological relationship has been established [119]. These wide ranging pro-inflammatory effects of PR3 in other conditions therefore may be also relevant in the pathophysiology of COPD, both directly by tissue damage and indirectly through other multiple pathways of inflammation.

## PR3 as a therapeutic target in COPD

There is considerable theoretical evidence and cell-based and animal-model data to support the role of PR3 in the development of COPD. However, as yet, PR3 activity in COPD has been poorly characterised.

To study PR3 in COPD requires the ability to quantify active (uninhibited) PR3 accurately and distinguish it from other NSPs to determine its specific function within biological samples. Reagents for free PR3 activity have only lately become available and until recently, detection required immunofluorescent staining of biopsy specimens, which if positive was followed by a PR3-ANCA specific enzyme-linked immunosorbent assay (ELISA) [120, 121]. Indeed, this was the internationally accepted method for diagnosing PR3-ANCA. Direct PR3 assays have been proposed as a biomarker to determine PR3 presence and production for assisting a diagnosis [122, 123]; however, like immunofluorescence techniques, they do not distinguish the active PR3 from PR3 which has been inactivated by its inhibitors. A similar challenge was seen for the measurement of NE activity and a novel approach to this has been the development of NE specific footprint, which may also be a more relevant approach for PR3 activity in vivo [124].

Whilst there is increasing interest in modifying NSP activity in conditions which predominantly feature neutrophilic inflammation, these have primarily focused on reducing the activity of NE and PR3 has not generally been considered as a relevant target in COPD.

The detection of PR3 activity, directly or indirectly, would improve our understanding of its role in COPD and individual patient’s disease activity. It would also potentially allow earlier diagnosis of diseases where PR3 activity was relevant (including COPD) before extensive damage has occurred. Understanding the role of PR3, might therefore allow earlier interventions and therapeutic strategies to be developed with PR3 as a valid target in COPD. Specific inhibitors might serve to reduce disease severity, mortality and the long-term health burdens of COPD. However, clearly the limited data available indicates there is much work to be done to clarify the likely relevance and hence impact of an anti-PR3 strategy.

## Conclusions

PR3 has many important functions that are relevant to human physiology and PR3 dysfunction may play a critical role in many processes central to the pathophysiology of COPD and other chronic neutrophilic human diseases. PR3 is the most abundant serine proteinase in the neutrophil, secondarily inhibited to NE, and, in addition to the role in general inflammation, PR3 can also cause direct tissue damage central to structural aspects of diseases such as COPD. This is consistent with the potential for PR3 to produce all the pathological changes of COPD that have traditionally been attributed to NE. Understanding this role and the impact on the inflammatory cascade has major implications for the design of anti-proteinase molecules aimed at restoring proteinase/anti-proteinase balance, ensuring that destructive activity of relevant serine proteinase action and amplification of inflammation is effectively limited, and thereby preventing the development and progression of COPD.

## Abbreviations

AAT: α-1 Anti-trypsin
AATD: α-1 Anti-trypsin Deficiency
ANCA: Anti-neutrophil Cytoplasmic Antibodies
C5a: Complement Component 5a
CF: Cystic Fibrosis
COPD: Chronic Obstructive Pulmonary Disease
COX: Cyclooxygenases
D_LCo_: Diffusing Capacity of the Lungs for Carbon Monoxide
ECM: Extracellular Matrix
ELISA: Enzyme-linked Immunosorbent Assay
GPA: Granulomatosis with Polyangiitis
GRN: Granulin
hCAP-18: Human Cathelicidin
IFN: Interferon
IL: Interleukin
NE: Neutrophil Elastase
NETs: Neutrophil Extracellular Traps
NFkB: Nuclear Factor Kappa-Light-Chain-Enhancer of Activated B Cells
NSP: Neutrophil Serine Proteinase
PGRN: Progranulin
PLSCR1: Phospholipid Scramblase 1
PR3: Proteinase 3
RAGE: Receptor for Advanced Glycation End-products
Serpins: Serine Proteinase Inhibitors
SPLI: Secretory Leukocyte Proteinase Inhibitor
Th: T helper Cells
TNF: Tumour Necrosis Factor

## References

[1] GOLD (2017). Global strategy for the diagnosis, management and prevention of chronic obstructive pulmonary disease.

[2] Baici A, Szedlacsek SE, Fruh H, Michel BA (1996). pH-dependent hysteretic behavior of human Myeloblastin (leucocyte proteinase 3). Biochem J.

[3] Karatepe K, Luo HR (2015). Proteinase 3 is expressed in stem cells and regulates bone marrow hematopoiesis. Blood.

[4] Korkmaz B, Moreau T, Gauthier F (2008). Neutrophil elastase, proteinase 3 and Cathepsin G: physiochemical properties, activity and Physiopathologcal functions. Biochemie.

[5] Zimmer M, Medcalf RL, Fink TM, Mattmann C, Lichter P, Jenne DE (1992). Three human elastase-like genes Coordingately expressed in the Myelomonocyte lineage are organised as a single genetic locus on 19pter. Proc Natl Acad Sci U S A.

[6] Csernok E, Ernst M, Schmitt W, Bainton DF, Gross WL (1994). Activated neutrophils express proteinase 3 on their plasma membrane *In vitro* and *In vivo*. Clin Exp Immunol.

[7] Witko-Sarsat V, Cramer EM, Hieblot C, Guichard J, Nusbaum P, Lopez S, Lesavre P, Halbwachs-Mecarelli L (1999). Presence of proteinase 3 in secretory vesicles: evidence of a novel, highly Mobilizable intracellular Pool distinct from Azurophil granules. Blood.

[8] Halbwachs-Mecarelli L, Bessou G, Lesavre P, Lopez S, Witko-Sarsat V (1995). Bimodal Distrubution of proteinase 3 (PR3) surface expression reflects a constitutive heterogeneity in the Polymorphonuclear neutrophil Pool. FEBS Lett.

[9] Csernok E, Ludemann J, Gross WL, Bainton DF (1990). Ultrastructural localisation of proteinase 3, the target antigen of anti-cytoplasmic antibodies circulating in Wegener's granulomatosis. Am J Pathol.

[10] Korkmaz B, Jaillet J, Jourdan M-L, Gauthier A, Gauthier F, Attucci S (2009). Catalytic activity and inhibition of Wegener antigen proteinase 3 on the cell surface of human Polymorphonuclear neutrophils. J Biol Chem.

[11] Korkmaz B, Lesner A, Letast S, Mahdi YK, Jourdan ML, Dallet-Choisy S, Marchand-Adam S, Kellenberger C, Viaud-Massuard MC, Jenne DE, Gauthier F (2013). Neutrophil proteinase 3 and dipeptidyl peptidase I (cathepsin C) as pharmacological targets in granulomatosis with polyangiitis (Wegener granulomatosis). Semin Immunopathol.

[12] Hajjar E, Broemstrup T, Kantari C, Witko-Sarsat V, Reuter N (2010). Structures of human proteinase 3 and neutrophil elastase - so similar yet so different. FEBS J.

[13] Hajjar E, Korkmaz B, Gauthier F, Brandsdal BO, Witko-Sarsat V, Reuter N (2006). Inspection of the binding sites of proteinase 3 for the Design of a Highly Specific Substrate. J Med Chem.

[14] Guarino C, Gruba N, Grzywa R, Dyguda-Kazimierowicz E, Hamon Y, Legowska M, Skorenski M, Dallet-Choisy S, Marchand-Adam S, Kellenberger C (2018). Exploiting the S4-S5 specificity of human neutrophil proteinase 3 to improve the potency of peptidyl Di(chlorophenyl)-phosphonate Ester inhibitors: a kinetic and molecular modeling analysis. J Med Chem.

[15] Fujinaga M, Chernaia MM, Halenbeck R, Koths K, James MNG (1996). The crystal structure of PR3, a neutrophil serine proteinase antigen of Wegener's granulomatosis antibodies. J Mol Biol.

[16] Korkmaz B, Horwitz MS, Jenne DE, Gauthier F (2010). Neutrophil elastase, proteinase 3, and Cathepsin G as therapeutic targets in human diseases. Pharmacol Rev.

[17] Rao NV, Wehner NG, Marshall BC, Gray WR, Gray BH, Hoidal JR (1991). Characterisation of proteinase 3 (PR-3), a neutrophil serine proteinase: structural and functional properties. J Biol Chem.

[18] Brubaker MJ, Groutas WC, Hoidal JR, Rao NV (1992). Human neutrophil proteinase 3: mapping of the substrate binding site using peptidyl Thiobenzyl esters. Biochem Biophys Res Commun.

[19] Schechter I, Berger A (1967). On the size of the active site in proteases. I. Papain. Biochem Biophys Res Commun.

[20] Twigg MS, Brockbank S, Lowry P, FitzGerald SP, Taggart C, Weldon S (2015). The role of serine proteases and Antiproteases in the cystic fibrosis lung. Mediat Inflamm.

[21] Berman HM, Westbrook J, Feng Z, Gilliland G, Bhat TN, Weissig H, Shindyalov IN, Bourne PE (2000). The Protein Data Bank. Nucleic Acids Res.

[22] Krieger E, Vriend G (2014). YASARA view - molecular graphics for all devices - from smartphones to workstations. Bioinformatics.

[23] Campbell EJ, Campbell MA, Owen CA (2000). Bioactive proteinase 3 on the cell surface of human neutrophils: quantification, catalytic activity, and susceptibility to inhibition. J Immunol.

[24] Loison F, Xu Y, Luo HR (2014). Proteinase 3 and serpin B1: a novel pathway in the regulation of Caspase-3 activation, neutrophil spontaneous apoptosis, and inflammation. Inflamm Cell Signal.

[25] Zani ML, Nobar SM, Lacour SA, Lemoine S, Boudier C, Bieth JG, Moreau T (2004). Kinetics of the inhibition of Neutriophil proteinases by recombinant Elafin and pre-elafin (Trappin-2) expressed in Pichia pastoris. Eur J Biochem.

[26] Yang L, Mei Y, Fang Q, Wang J, Yan Z, DSong Q, Lin Z, Ye G. Identification and characterization of serine protease inhibitors in a parasitic wasp, Pteromalus puparum. Sci Rep. 2017;7:1-13. 10.1038/s41598-017-16000-5 PMC5691223 29147019

[27] Sinden NJ, Baker MJ, Smith DJ, Kreft J-U, Dafforn TR, Stockley RA (2015). Alpha-1-antitrypsin variants and the proteinase/anti-proteinase imbalance in chronic obstructive pulmonary disease. Am J Physiol Lung Cell Mol Physiol.

[28] Jerke U, Perez Hernandez D, Beaudette P, Korkmaz B, Dittmar G, Kettritz R (2015). Neutrophil Serine Proteases Exert Proteolytic Activity on Endothelial Cells. Kidney Int.

[29] Korkmaz B, Lesner A, Guarino C, Wysocka M, Kellenberger C, Watier H, Specks U, Gauthier F, Jenne DE (2016). Inhibitors and antibody fragments as potential anti-inflammatory therapeutics Targetting neutrophil proteinase 3 in human disease. Pharmacol Rev.

[30] Guyot N, Wartelle J, Malleret L, Todorov AA, Devouassoux G, Pacheco Y, Jenne DE, Belaaouaj A (2014). Unopposed Cathepsin G, neutrophil elastase, and proteinase 3 cause severe lung damage and emphysema. Am J Pathol.

[31] Joosten LA, Netea MG, Fantuzzi G, Koenders MI, Helsen MM, Sparrer H, Pham CT, van der Meer JW, Dinarello CA, van den Berg WB (2009). Inflammatory Arthritis in Caspase 1 Gene-deficient Mice: Contribution of Proteinase 3 to Caspase 1-independent Production of Bioactive Interleukin-1beta. Arthritis Rheum.

[32] Kessenbrock K, Frohlich L, Sixt M, Lammermann T, Pfister H, Bateman A, Belaaouaj A, Ring J, Ollert M, Fassler R, Jenne DE (2008). Proteinase 3 and neutrophil elastase enhance inflammation in mice by inactivating anti-inflammatory Progranulin. J Clin Invest.

[33] Gudmann Natasja Stæhr, Manon-Jensen Tina, Sand Jannie Marie Bülow, Diefenbach Claudia, Sun Shu, Danielsen Annette, Karsdal Morten Asser, Leeming Diana Julie (2018). Lung tissue destruction by proteinase 3 and cathepsin G mediated elastin degradation is elevated in chronic obstructive pulmonary disease. Biochemical and Biophysical Research Communications.

[34] Newby PR, Carter RI, Stockley RA. Aα-Val^541^ a Novel Biomarker of Proteinase 3 Activity. Am J Respir Crit Care Med. 2017;195:A5247.

[35] Newby PR, Stockley RA. Neutrophil elastase and proteinase 3 activity in PiSZ Alpha-1 antitrypsin deficiency. Am J Respir Crit Care Med. 2018;197:A4763.

[36] Hoenderdos K, Condliffe A (2013). The neutrophil in chronic obstructive pulmonary disease. Too little, too late or too much, too soon?. Am J Respir Cell Mol Biol.

[37] Campbell EJ, Campbell MA, Boukedes SS, Owen CA (2000). Quantum proteolysis by neutrophils: implications for pulmonary emphysema in α1-antitrypsin deficiency. CHEST.

[38] Stockley RA (1999). Neutrophils and protease/Antiprotease imbalance. Am J Respir Crit Care Med.

[39] Owen CA, Campbell EJ (1999). The cell biology of leukocyte-mediated proteolysis. J Leukoc Biol.

[40] Maximova K, Venken T, Reuter N, Trylska J (1860). D-peptides as inhibitors of PR3-membrane interactions. Biomembranes.

[41] Korkmaz B, Poutrain P, Hazouard E, de Monte M, Attucci S, Gauthier FL (2005). Competition between elastase and related proteases from human neutrophil for binding to α1-protease inhibitor. Am J Respir Cell Mol Biol.

[42] Sinden NJ, Stockley RA (2013). Proteinase 3 activity in sputum from subjects with Alpha-1 anti-trypsin deficiency and COPD. Eur Respir J.

[43] Kao RC, Wehner NG, Skubitz KM, Gray BH, Hoidal JR (1988). Proteinase 3. A distinct human Polymorphonuclear leukocyte proteinase that produces emphysema in hamsters. J Clin Invest.

[44] Borel Florie, Sun Huaming, Zieger Marina, Cox Andrew, Cardozo Brynn, Li Weiying, Oliveira Gabriella, Davis Airiel, Gruntman Alisha, Flotte Terence R., Brodsky Michael H., Hoffman Andrew M., Elmallah Mai K., Mueller Christian (2018). Editing out fiveSerpina1paralogs to create a mouse model of genetic emphysema. Proceedings of the National Academy of Sciences.

[45] Lombard C, Bouchu D, Wallach J, Saulnier J (2005). Proteinase 3 hydrolysis of peptides derived from human elastin exon 24. Amino Acids.

[46] Chelladurai P, Seeger W, Pullamsetti SS (2012). Matrix metalloproteinases and their inhibitors in pulmonary hypertension. Eur Respir J.

[47] Sand JMB, Knox AJ, Lange P, Sun S, Kristensen JH, Leeming DJ, Karsdal MA, Bolton CE, Johnson SR (2015). Accelerated extracellular matrix turnover during exacerbations of COPD. Respir Res.

[48] Ramaha A, Patston PA (2002). Release and degradation of angiotensin 1 and angiotensin 2 from angiotensinogen by neutrophil serine proteinases. Arch Biochem Biophys.

[49] Witko-Sarsat V, Halbwachs-Mecarelli L, Schuster A, Nusbaum P, Ueki I, Canteloup S, Lenoir G, Descamps-Latscha B, Nadel JA (1999). Proteinase 3, a potent Secretagogue in airways, is present in cystic fibrosis sputum. Am J Respir Cell Mol Biol.

[50] Janciauskiene S, Bals R, Koczulla R, Vogelmeier C, Kohnlein T, Welte T (2011). The discovery of Alpha-1-antitrypsin and its role in health and disease. Respir Med.

[51] Sorensen OE, Follin P, Johnsen AH, Calafat J, Tjabringa GS, Hiemstra PS, Borregaard N (2001). Human cathelicidin, hCAP-18, is processed to the antimicrobial peptide LL-37 by extracellular cleavage with proteinase 3. Blood J.

[52] Ren K, Torres R (2009). Role of interleukin-1beta during pain and inflammation. Brain Res Rev.

[53] Popa C, Netea MG, van Riel PLCM, van der Meer JWM, Stalenhoef AFH (2007). The role of TNF-a in chronic inflammatory conditions, intermediary metabolism, and cardiovascular risk. J Lipid Res.

[54] Coeshott C, Ohnemus C, Pilyavskaya A, Ross S, Wieczorek M, Kroona H, Leimer AH, Cheronis J (1999). Converting enzyme-independent release of tumor necrosis factor alpha and IL-1beta from a stimulated human Monocytic cell line in the presence of activated neutrophils or purified proteinase 3. Proc Natl Acad Sci U S A.

[55] Keyel Peter A. (2014). How is inflammation initiated? Individual influences of IL-1, IL-18 and HMGB1. Cytokine.

[56] Schreiber A, Pham CT, Hu Y, Schneider W, Luft FC, Kettritz R (2012). Neutrophil serine proteases promote IL-1beta generation and injury in necrotizing crescentic glomerulonephritis. J Am Soc Nephrol.

[57] Sugawara A, Uehara A, Nochi T, Yamaguchi T, Ueda H, Sugiyama A, Hanzawa K, Kumagai K, Okamura H, Takada H (2001). Neutrophil proteinase 3-mediated induction of bioactive IL-18 secretion by human Oral epithelial cells. J Immunol.

[58] Bradley JR (2008). TNF-mediated inflammatory disease. J Pathol.

[59] Hurst SM, Wilkinson TS, McLoughline RM, Jones S, Horiuchi S, Yamamoto N, Rose-john S, Fuller GM, Topley N, Jones SA (2001). IL-6 and its soluble receptor Ochestrate a Temportal switch in the pattern of leukocyte recruitment seen during acute inflammation. Immunity.

[60] McLoughlin RM, Hurst SM, Nowell MA, Harris DA, Horiuchi S, Morgan LW, Wilkinson TS, Yamamoto N, Topley N, Jones SA (2004). Differential regulation of neutrophil-activating chemokines by IL-6 and its soluble receptor isoforms. J Immunol.

[61] Keatings VM, Collins PD, Scott DM, Barnes PJ (1996). Differences in Interleukin-8 and tumour necrosis factor-alpha in induced sputum from patients with chronic obstructive pulmonary disease or asthma. Am J Respir Crit Care Med.

[62] Rani L, Minz RW, Sharma A, Anand S, Gupta D, Panda NK, Sakhuja VK (2015). Predominance of PR3 specific immune response and skewed Th17 vs. T-regulatory Miliew in active Granfulomatosis with Polyangiitis. Cytokine.

[63] Kim S-H, Han S-Y, Azam T, Yoon D-Y, Dinarello CA (2005). Interleukin-32: a cytokine and inducer of TNFα. Immunity.

[64] Calabrese F, Baraldo S, Bazzan E, Lunardi F, Rea F, Maestrelli P, Turato G, Lokar-Oliani K, Papi A, Zuin R, Sfriso P (2008). IL-32, a novel Proinflammatory cytokine in chronic obstructive pulmonary disease. Am J Respir Crit Care Med.

[65] Zhang J-M, An J (2007). Cytokines, inflammation and pain. Int Anesthesiol Clin.

[66] Nemeth T, Mocsai A (2016). Feedback amplification of neutrophil function. Cell.

[67] Robache-Gallea S, Morand V, Bruneau JM, Schoot B, Tagat E, Realo E, Chouaib S, Roman-Roman S (1995). In vitro processing of human tumour necrosis factor-alpha. J Biol Chem.

[68] Kessenbrock K, Dau T, Jenne DE (2011). Tailor-made inflammation: how neutrophil serine proteases modulate the inflammatory response. J Mol Med.

[69] Ungers MJ, Sinden NJ, Stockley RA (2014). Progranulin is a substrate for neutrophil-elastase and Proteinase-3 in the airway and its concentration correlates with mediators of airway inflammation in COPD. Am J Physiol Lung Cell Mol Physiol.

[70] Couto MA, Harwig SSL, Cullor JS, Hughes JP, Lehrer RI (1992). eNAP-2, a novel cysteine-rich bactericidal peptide from equine leukocytes. Infect Immun.

[71] Baker M, Mackenzie IR, Pickering-Brown SM, Gass J, Rademakers R, Lindholm C, Snowden J, Adamson J, Sadovnivk AD, Rollinson S (2006). Mutations in Progranulin cause tau-negative frontotemporal dementia linked to chromosome 17. Nature.

[72] Zhu J, Nathan C, Jin W, Sim D, Ashcroft GS, Wahl SM, Lacomis L, Erdujument-Bromage H, Tempst P, Wright CD, Ding A (2002). Conversion of Proepithelin to Epithelins: roles of SLPI and elastase in host defense and wound repair. Cell.

[73] van der Berg CW, Tambourgi DV, Clark HW, Hoong SJ, Spiller OB, McGreal EP (2014). Mechanism of neutrophil dysfunction: neutrophil serine proteases cleave and inactivate the C5a receptor. J Immunol.

[74] Marc MM, Korosec P, Kosnik M, Kern I, Flezar M, Suskovic S, Sorli J (2004). Complement factors C3a, C4a, and C5a in chronic obstructive pulmonary disease and asthma. Am J Respir Cell Mol Biol.

[75] Zhang Y, Jiang Y, Sun C, Wang Q, Yang Z, Pan X, Zhu M, Xiao W (2014). The human cathelicidin LL-37 enhances airway mucus production in chronic obstructive pulmonary disease. Biochem Biophys Res Commun.

[76] Campanelli D, Detmers PA, Nathan CF, Gabay JE (1990). Azurocidin and a homologous serine protease from neutrophils. Differential antimicrobial and proteolytic properties. J Clin Invest.

[77] Dasaraju PV, Liu C. Infections of the respiratory system. In: Medical Microbiology. 4th ed. Baron S, editor. Texas: University of Texas Medical Branch at Galveston; 1996. 21413304

[78] Kuroda K, Okumura K, Isogai H, Isogai E (2015). The human cathelicidin antimicrobial peptide LL-37 and mimics are potential anti-Cancer drugs. Front Oncol.

[79] Jiang Yuan-Yuan, Xiao Wei, Zhu Mao-Xiang, Yang Zhi-Hua, Pan Xiu-Jie, Zhang Yi, Sun Cong-Cong, Xing Ying (2012). The effect of human antibacterial peptide LL-37 in the pathogenesis of chronic obstructive pulmonary disease. Respiratory Medicine.

[80] Urban CF, Ermert D, Schmid M, Abu-Abed U, Goosmann C, Nacken W, Brinkmann V, Jungblut PR, Sycglinsky A (2009). Neutrophil extracellular traps contain calprotectin, a cytosolic protein complex involved in host defense against Candida albicans. PLoS Pathog.

[81] Kessenbrock K, Krumbholz M, Schonermarck U, Back W, Gross WL, Werb Z, Grone H-J, Brinkmann V, Jenne DE (2009). Netting Neutrophils in Autoimmune Small-Vessel Vasculitis. Nat Med.

[82] Delgado-Rizo V, Martinez-Guzman MA, Iniguez-Gutierrez L, Garcia-Orozco A, Alvarado-Navarro A, Fafutis-Morris M (2017). Neutrophil extracellular traps and its implications in inflammation: an overview. Front Immunol.

[83] Beiter K, Wartha F, Albiger B, Normark S, Zychlinsky A, Henriques-Normark B (2006). An endonuclease allows *Streptococcus pneumoniae* to escape from neutrophil extracellular traps. Curr Biol.

[84] Hong W, Juneau RA, Pang B, Swords WE (2009). Survival of bacterial biofilms within neutrophil extracellular traps promotes Nontypeable *Haemophilus influenzae* persistence in the Chinchilla model for otitis media. J Innate Immun.

[85] Benarafa C, Priebe GP, Remold-O'Donnell E (2007). The neutrophil serine protease inhibitor SerpinB1 preserves lung Defence functions in *Pseudomonas aeruginosa* infection. J Exp Med.

[86] Rao Narayanam V., Marshall Bruce C., Gray Beulah H., Hoidal John R. (1993). Interaction of Secretory Leukocyte Protease Inhibitor with Proteinase-3. American Journal of Respiratory Cell and Molecular Biology.

[87] Kasahara Y, Tuder RM, Cool CD, Lynch DA, Flores SC, Voelkel NF (2001). Endothelial cell death and decreased expression of vascular endothelial growth factor and vascular endothelial growth factor receptor 2 in emphysema. Am J Respir Crit Care Med.

[88] Kolonin AG, Sergeeva A, Staquicini DI, Smith TL, Tarleton CA, Molldrem JJ, Sidman RL, Marchio S, Pasqualini R, Arap W (2017). Interaction between tumour cell surface receptor RAGE and proteinase 3 mediates prostate Cancer Matastasis to bone. Cancer Res.

[89] Sukkar MB, Ullah MA, Gan WJ, Wark PAB, Chung KF, Hughes JM, Armour CL, Phipps S (2012). RAGE: a new frontier in chronic airways disease. Br J Pharmacol.

[90] Yonchuk JG, Silverman EK, Bowler RP, Agusti A, Lomas DA, Miller BE, Tal-Singer R, Mayer RJ (2015). Circulating soluble receptor for advanced glycation end products (sRAGE) as a biomarker of emphysema and the RAGE Axis in the lung. Am J Respir Crit Care Med.

[91] Stehlik C (2009). Multiple IL-1β converting enzymes contribute to inflammatory arthritis. Arthritis Rheum.

[92] Lee T-H, Song HJ, Park C-S (2014). Role of Inflammasome activation in development and exacerbation of asthma. Asia Pacific Allergy.

[93] Toonen EJ, Mirea AM, Tack CJ, Stienstra R, Ballak DB, van Diepen JA, Hijmans A, Chavakis T, Dokter WH, Pham CT (2016). Activation of proteinase 3 contributes to non-alcoholic fatty liver disease (NAFLD) and insulin resistance. Mol Med.

[94] Gracie JA (2004). Interleukin-18 as a potential target in inflammatory arthritis. Clin Exp Immunol.

[95] Matsumoto Takeshi, Kaneko Toshihiro, Seto Masashi, Wada Hideo, Kobayashi Toshihiko, Nakatani Kaname, Tonomura Harue, Tono Yasutaka, Ohyabu Mariko, Nobori Tsutomu, Shiku Hiroshi, Sudo Akihiro, Uchida Atsumasa, Stearns Kurosawa Deborah J., Kurosawa Shinichiro (2008). The Membrane Proteinase 3 Expression on Neutrophils Was Downregulated After Treatment With Infliximab in Patients With Rheumatoid Arthritis. Clinical and Applied Thrombosis/Hemostasis.

[96] Armstrong L, Godinho SIH, Uppington KM, Whittington HA, Millar AB (2009). Tumour necrosis factor-α processing in interstitial lung disease: a potential role for exogenous Proteinase-3. Clin Exp Immunol.

[97] McGreal EP, Davies PL, Powell W, Rose-John S, Spiller OB, Doull I, Jones SA, Kotecha S (2010). Inactivation of IL-6 and soluble IL-6 receptor by neutrophil derived serine proteases in cystic fibrosis. Biochim Biophys Acta.

[98] Dinarello CA, Kim SH (2006). IL-32, a novel cytokine with a possible role in disease. Ann Rheum Dis.

[99] Zhao Y-P, Tian Q-Y, Liu C-J (2013). Progranulin deficiency exaggerates, whereas Progranulin-derived Atsttrin attenuates, severity of dermatitis in mice. FEBS Lett.

[100] Tang W, Lu Y, Tian QY, Zhang Y, Guo FJ, Liu GY, Syed NM, Lai Y, Lin EA, Kong L (2011). The growth factor Progranulin binds to TNF receptors and is therapeutic against inflammatory arthritis in mice. Science.

[101] Guo Z, Li Q, Han Y, Liang Y, Xu Z, Ren T (2012). Prevention of LPS-induced acute lung injury in mice by Progranulin. Mediat Inflamm.

[102] Goedert M, Spillantini MG (2006). Frontotemporal lobar degeneration through loss of Progranulin function. Brain Res Rev.

[103] Bossu P, Salani F, Alberici A, Archetti S, Bellelli G, Galimberti D, Scarpini E, Spalletta G, Caltagirone C, Padovani A, Borroni B (2011). Loss of function mutations in the Progranulin gene are related to pro-inflammatory cytokine dysregulation in frontotemporal lobar degeneration patients. J Neuroinflammation.

[104] Cruts M, van Broeckhoven C (2008). Loss of Progranulin function in frontotemporal lobar degeneration. Trends Genet.

[105] Chow MJ, Choi M, Yun SH, Zhang Y (2013). The effect of static stretch on elastin degradation in arteries. PLoS One.

[106] Kuckleburg CJ, Tilkens SM, Santoso S, Newman PJ (2012). Proteinase 3 contributes to Transendothelial migration of NB1-positive neutrophils. J Immunol.

[107] Wiedow O, Meyer-Hoffert U (2005). Neutrophil serine proteases: potential key regulators of cell Signalling during inflammation. J Intern Med.

[108] Saragih H, Zilian E, Jaimes Y, Paine A, Figueiredo C, Eiz-Vesper B, Blascysk R, Larmann J, Theilmeier G, Burg-Roderfeld M (2014). PECAM-1-dependent Heme Oxygenase-1 regulation via an Nrf2-mediated pathway in endothelial cells. Thromb Haemost.

[109] Lutz PG, Houzel-Charavel A, Moog-Lutz C, Cayre YE (2001). Myeloblastin is an Myb target gene: mechanisms of Regulationin myeloid leukemia cells growth-arrested by retinoic acid. Blood Journal.

[110] Lutz PG, Moog-Lutz C, Coumau-Gatbois E, Kobari L, di Gioia Y, Cayre YE (2000). Myeloblastin is a granulocyte Colony-stimulating factor-responsive gene conferring factor-independent growth to Haematopoietic cells. Proc Natl Acad Sci U S A.

[111] Bories D, Raynal M-C, Solomon DH, Darzynkiewicz Z, Cayre YE (1989). Down-regulation of a serine protease, Myeloblastin, causes growth arrest and differentiation of Promyelocytic leukemia cells. Cell.

[112] Martin KR, Kantarl-Mimoun C, Yin M, Perderzoll-Ribell M, Angelot-Delettre F, Cerol A, Grauffel C, Benhamou M, Reuter N, Saas P (2016). Proteinase 3 is a phosphatidylserine-binding protein that affects the production and function of microvesicles. J Biol Chem.

[113] Cerezo LA, Kuklova M, Hulejova H, Vernerova Z, Kasprikova N, Veigl D, Pavelka K, Vencovsky J, Senolt L (2015). Progranulin is associate with disease activity in patients with Rheumatiod arthritis. Mediat Inflamm.

[114] Jennette JC, Nachman PH (2017). ANCA glomerulonephritis and Vasculitis. Clin J Am Soc Nephrol.

[115] Hozumi H, Enomoto N, Oyama Y, Kono M, Fujisawa T, Inui N, Nakamura Y, Suda T (2016). Clinical implication of Proteinase-3-Antineutrophil cytoplasmic antibody in patients with idiopathic interstitial pneumonias. Lung.

[116] Seo P, Stone JH (2004). The Antineutrophilic cytoplasmic antibody-associated Vasulitides. Am J Med.

[117] Falk RJ, Jennette JC (1991). Wegener's granulomatosis systemic Vasculitis, and Antineutrophil cytoplasmic autoantibodies. Annu Rev Med.

[118] Savage COS (2011). Pathogenesis of anti-neutrophil cytoplasmic autoantibody (ANCA)-associated Vasculitis. Clin Exp Immunol.

[119] Bonarius HPJ, Brandsma CA, Kerstjens HAM, Koerts JA, Kerkhof M, Nizankowska-Mogilnicka E, Roozendaal C, Postma DS, Timens W (2011). Antinuclear autoantibodies are more prevalent in COPD in association with low body mass index but not with smoking history. Thorax.

[120] Savige J, Gillis D, Benson E, Davies D, Esnault V, Falk RJ, Hagen EC, Jayne D, Jennette JC, Paspaliaris B (1999). International consensus statement on testing and reporting of Antineutrophil cytoplasmic antibodies (ANCA). Am J Clin Pathol.

[121] Savige J, Dimech W, Fritzler M, Goeken J, Hagen EC, Jennette JC, McEvoy R, Pusey C, Pollock W, Trevisin M (2003). Addendum to the international consensus statement on testing and reporting of Antineutrophil cytoplasmic antibodies. Am J Clin Pathol.

[122] Wong HR, Cvijanovich NZ, Anas N, Allen GL, Thomas NJ, Bigham MT, Weiss SL, Fitzgerald J, Checchia PA, Meyer K (2015). A multibiomarker-based model for estimating the risk of septic acute kidney injury. Crit Care Med.

[123] Ng LL, Khan SQ, Narayan H, Quinn P, Squire IB, Davies JE (2011). Proteinase 3 and prognosis of patients with acute myocardial infarction. Clin Sci.

[124] Carter R. I., Ungurs M. J., Mumford R. A., Stockley R. A. (2012). A -Val360: a marker of neutrophil elastase and COPD disease activity. European Respiratory Journal.

